# The impact of music listening intervention on Asia elderly with dementia: a systematic review

**DOI:** 10.1186/s40001-023-01355-5

**Published:** 2023-11-21

**Authors:** Chen Lin, He Xuanxu, Xue Yuyang, Xu Zhongqiu, Chunhai Gao

**Affiliations:** 1 Guangzhou Sport University, Guangzhou, Guangdong Province China; 2 Faculty of Education, Shenzhen University, Nanhai Blvd, Nanshan, Shenzhen, 518060 Guangdong Province China

**Keywords:** Dementia, Music therapy, China, older adults, Mini-Mental State Examination

## Abstract

From an initial pool of 2303 studies, ten eligible and potential studies were selected through rigor inclusion and exclusion criteria for this systematic review to examine music therapy's effect on dementia. The review included 967 participants, with the majority being female. A significant number of studies were conducted in Taiwan. Although several cognitive ability assessment methods were employed in the selected studies, the Mini-Mental State Examination (MMSE) was the most commonly used tool for evaluating the effects of music therapy on dementia. Overall, the current review demonstrates that music therapy can be a valuable strategy for treating patients with dementia, with its outcomes including improved cognitive function and potentially slowing the progression of the disease's severity. Therefore, this study can significantly contribute to future studies and practices aimed at using music therapy to treat dementia.

## Introduction

Dementia is a general term that describes diseases and conditions marked by a gradual decline in cognitive abilities, such as memory and language, as well as changes in behavior that may include depression and anxiety [1, 2]. It is a neurodegenerative disorder characterized by a decline in cognitive function, affecting memory, thinking, behavior, and emotion. The recent World Health Organization (WHO) report showed that more than 55 million people worldwide are currently affected by dementia [3]. According to this report, age—most commonly 65 years or older—increases the risk of developing dementia. Hence, with an aging population, the prevalence of dementia is expected to rise, creating significant challenges for healthcare systems worldwide. As China has undergone social and economic development in recent decades, its population has rapidly aged, leading to a proliferation of neurodegenerative disorders such as dementia [4].

It is a common trend to treat patients with dementia via pharmacological as well as non-pharmacological approaches. However, the pharmaceutical approaches cause side effects, including sedation, falls and extrapyramidal signs, reduced well-being and quality of life, increased cognitive decline rate, and more [5]. To make matters worse, pharmaceutical intervention for older adults may be even more challenging. Therefore, it is crucial at this point to explore alternative non-pharmaceutical approaches for treating dementia. Music therapy has been employed for several years to help alleviate symptoms of dementia [6, 7]. This technique can enhance mood, cognitive functions, and memory while providing a sense of connection and socialization for patients experiencing isolation [7, 8].

Despite music therapy being considered a promising intervention for people with dementia, the results of previous studies have shown discrepancies. While some studies suggest positive outcomes, others report inconclusive or adverse effects. Therefore, a systematic review is necessary to summarize and evaluate the existing evidence on the effectiveness of music therapy against dementia in older adults. We searched scientific literature using well-known databases to gather recent studies on the effect of music on dementia. After applying appropriate screening criteria, we selected the necessary number of studies for analysis in the current systematic review. This review will provide an overview of the current state of research, highlight potential gaps in knowledge, and offer recommendations for future studies and clinical practice.

## Methods

### Literature search

To examine the literature, we used PRISMA (Preferred Reporting Items for Systematic Reviews and Meta-analyses) guidelines, the recommended standards for conducting systematic reviews [9, 10]. The PRISMA guidelines involve three planning phases: study identification, literature examination, and inclusion. We searched for articles about music therapy's effect on dementia based on our research objective.

The articles were searched against the well-known scientific literature repository databases, including Scopus, PubMed, and Web of Science. A combination of terms such as ("music," OR "music therapy," OR "music intervention," OR "music care") AND ("dementia" OR "Alzheimer's disease") was employed for the literature search. The literature search was conducted in March 2023.

### Study selection

The selection criteria for including articles in this systemic review are listed in Table 1. Accordingly, articles were screened considering the study topic, geographic area, participants' age, publication year, article type, and publication language. The details of each selection criteria can be referred to in Table 1.

**Table 1:** Inclusion and exclusion criteria to select studies for this systemic review

Criteria	Inclusion	Exclusion	Remark
Study focus topic	M.T. and dementia	Other therapies and disorders	Included if M.T. and dementia are combined with other topics
Study subject age group	Elderly	Other age groups	
Study area	China	Other geographic areas	Including Mainland and SAR
Publication year	2013–2023^a^	Before 2013	Including the initial and the final year
Study type	Full-length journal research articles	Reviews, conference papers, book chapters, non-full-length editorials	
Category of articles	Quantitative	Non-quantitative	Included quantitative and qualitative
Language	English	Non-English	

MT: music therapy; SAR: Special administrative regions (Taiwan, Hong Kong, and Macau)

^a^Studies published until March 2013

### Data extraction and organization

First, the studies that fulfill the criteria above to be included in this review were imported into the Endnote reference management software. The selected articles were organized orderly in serial numbers (Table 2) for further communications. Articles were arranged based on their year of publication from the earliest to the latest. The selected articles were studied thoroughly to find common extractable data to assess the intervention approaches and outcomes. These extractable data items should commonly be found in two or more selected studies. The extracted data were handled in the Excel spreadsheet.

**Table 2:** Basic information on selected studies and participants' age

S.N.	Ref.	Period		Place of study	Participants' age (year)
		Study conducted	Publication^a^		
1	[13]	–	2014	Taiwan	65–97
2	[14]	April 2008 to May 2010	2015	Taiwan	65–95
3	[15]	–	2017	Hangzhou	$\ge$ 60
4	[16]	March 2013 to November 2013	2017	Taiwan	$\ge$ 60
5	[17]	August 2014 to December 2016	2018	Beijing	$\ge$ 65
6	[18]	October 2012 to March 2013	2018	Taiwan	$\ge$ 55
7	[19]	–	2019	Hong Kong	85.3 ^b^
8	[20]	–	2020	Hong Kong	$\ge$ 65
9	[21]	–	2020	Hangzhou	$\ge$ 60
10	[22]	August 2020 to June 2021	2023	Taiwan	$\ge$ 65

MT: music therapy; M/F/T: male/female/total; AD: Alzheimer’s disease

^a^Publication issued year

^b^Mean age

### Quality assessment

In this review, the Mixed Methods Appraisal Tool (MMAT) version 2018 [11] was employed to evaluate the quality of the selected articles. To eliminate bias, the selected studies' quality was checked by two authors simultaneously. In this version of the MMAT, five different criteria for each category of study and two common screening questions are considered. The responses, including *yes*, *no*, or *cannot tell*, accompany these questions. The quality of the final articles to be included in this systematic review (Table 2) was evaluated and approved following these steps.

## Results

### Literature search

**Fig. 1:**
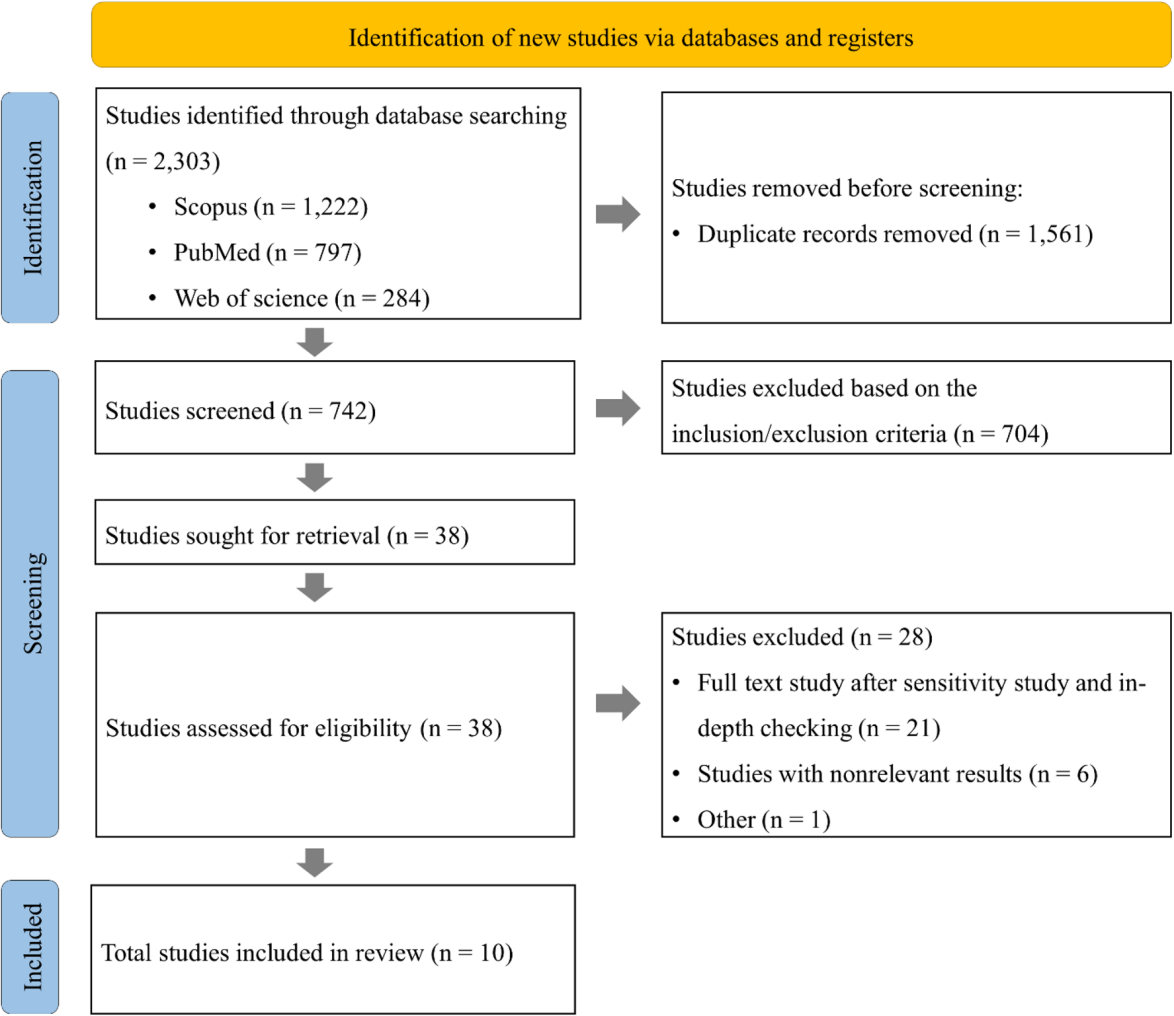
Flowchart showing literature search for this review; prepared using PRISMA

Literature was searched to collect studies on the effect of music therapy on dementia, as explained in the Methods section. The first search was conducted using the abovementioned terms without filtration. During this stage, 2303 studies were generated, of which 1222, 797, and 284 were from Scopus, PubMed, and Web of Science. As the largest abstract and citation research literature database, Scopus [12] comprised 53% of the total searched studies on this topic. The studies were filtered using the PRISMA approach. Nearly 68 and 31% of the literature were excluded from this review due to the replication of studies in the databases and using inclusion/exclusion criteria, respectively. The inclusion criteria employed in this review include (1) age group (elderly), (2) study geographic area (China), (3) article type (complete length journal research), (4) publication year (2013–2023), (5) article category (quantitative), and (6) language (English) (Table 1). Finally, ten studies were screened out to be included in this systemic review (Table 2). The detailed literature screening process is shown in Fig. 1.

### Information on the selected studies and participants

Table 2 organized selected studies in an orderly based on publication year and contained information on the study period and publication, places of study in China, and participants' ages. The earliest and the latest studies were published in 2014 and 2023, respectively. Half of the literature screened for this systemic review was studied in Taiwan. This review also included other studies, consisting of two from Beijing and Hong Kong and one from Hangzhou. Regarding the participant's age, most studies include ≥ 65 years old. Only one study (Table 2 #6) included participants ≥ 55 years old.

Information on the number of participants in the selected studies is shown in Table 3. The total number of participants allocated in the initial stage of the study was 967. However, nearly 10% of the participants discontinued the study process for various reasons. Most participants (~ 53% of the initially allocated participants) discontinued study #2. On the contrary, all the initially allocated participants in studies #7 and #10 could complete the experimentation. Study #5 and #6 included the highest and the lowest number of participants in their research, respectively. Therefore, it is essential to consider these scenarios when allocating study participants to avoid ending up with a statistically unacceptable sample size. All studies allocate experimental (music therapy) and control groups. There were more participants in the experimental group than in the control counterpart. Another interesting fact is that all the studies included in this review allocated a significantly higher number of female participants than males.

**Table 3:** Information on the number of participants

Study S.N.	Initial allocation			Completed the study				
	Music therapy	Control	Total	Music therapy		Control		Total
				M/F	Total	M/F	Total	
1	52	52	104	^a^	49	^a^	51	100
2	52	35	87	5/15	20	8/13	21	41
3	24	24	48	7/12	19	5/16	21	40
4	104	68	172	5/85	90	5/54	59	149
5	100	99	199	40/57	97	39/56	95	192
6	15	15	30	6/9	15	8/5	13	28
7	40	33	73	12/28	40	10/23	33	73
8	58	53	165	15/43	58^b^	13/40^d^	53^d^	165^e^
	54			12/42	54^c^			
9	22	22	44	6/15	21	5/17	22	43
10	25	20	45	4/21	25	5/15	20	45
Total	546	421	967		488		388	876

Out of the total 100 participants, 47 were male, and 53 were female

^a^The information is not available

^b^Music with movement;

^c^Was music listening

^d^Social activity

^e^Not all completed the intervention, but all participants were considered in the analysis

### Music therapy for dementia

Table 4 shows approaches, durations, and outcomes of music intervention to treat dementia. Various music therapy approaches, such as listening, singing, dancing, and playing instruments, were employed alone or in combination with other activities. Singing followed by listening was the most implemented music therapy approach. Singing or listening was employed in all the selected studies in Table 4. Generally, interventions were conducted up to 30–120 min/session for 6–24 weeks. Study #1 and #5, which conducted interventions for 30 and 30–40 min/session, twice/week, and twice/day for six weeks and three months, respectively, represent the shortest and the most extended intervention durations.

**Table 4:** Intervention approach and outcomes

Study S.N.	Approach	Duration	Outcome			
			Experimental group		Control group	
			Baseline	Follow up	Baseline	Follow up
1	Listening, singing, coloring sound bell, and other activities	30 min/session, twice/week for six weeks	CSDD = 17.39 ± 9.56^a^ MMSE = 12.80 ± 6.15	CSDD = 11.23 ± 8.64*** MMSE = 14.24 ± 6.39*	CSDD = 15.70 ± 10.16 MMSE = 13.76 ± 5.36	CSDD = 11.43 ± 9.72^ns^ MMSE = 13.50 ± 4.60^ns^
2	Receptive listening	30 min/day for six months	MMSE = 17.6 ± 5.98	MMSE = 16.7 ± 5.55^ ns^	MMSE = 14.7 ± 3.35	MMSE = 14.1 ± 3.71
3	Singing and other folk recreation activities	40–50 min/session, three Times/week for 16 weeks	MMSE = 14.58 ± 5.59^ns^	MMSE = 17.00 ± 4.03**	MMSE = 14.48 ± 4.40	MMSE = 13.05 ± 5.48
4	Listening and physical activity with music	30 min/session, twice/week for 24 weeks	CSDD = 3.088 ± 1.43** MMSE = 18.06 ± 8.24**	CSDD = 2.47 ± 0.79^ns^ MMSE = 16.29 ± 7.50*	CSDD = 1.10 ± 1.89 MMSE = 22.71 ± 6.47	CSDD = 1.16 ± 2.50 MMSE = 21.14 ± 5.23
5	Singing and/or listening	30–40 min/session, twice/day for three months	MMSE = 13.45 ± 3.66^ns^	MMSE = 13.34 ± 4.00^ns^	MMSE = 13.22 ± 4.01	MMSE = 12.98 ± 4.15
6	Walking while singing and playing an instrument	60 min/session, weekly for two months	MMSE = 16.4 ± 7.3* CMAI-C = 44.3 ± 14.8*	– CMAI-C = 41.9 ± 13.4**	MMSE = 17.9 ± 3.7 CMAI-C = 34.7 ± 10.9	– CMAI-C = 39.0 ± 15.7
7	Singing along or dancing to the music	120 min/session for 16 sessions	MMSE = 13.9 ± 5.8^ns^ Agitation = 3.4 ± 3.7^ns^	─ Agitation = 1.4 ± 2.0**	MMSE = 12.2 ± 6.2 Agitation = 2.1 ± 3.3	– Agitation = 1.8 ± 2.6
8	Listening, movement, dancing, playing instruments	30–45 min/session, twice/week for six weeks	MMSE = 10.99 ± 4.16^ ns^ CMAI-NH = 40.97 ± 20.04^ns^	– CMAI-NH = 33.07 ± 14.69***	MMSE = 11.97 ± 4.43 CMAI-NH = 37.20 ± 15.02	– CMAI-NH = 30.94 ± 11.41
			MMSE = 12.12 ± 4.13 CMAI-NH = 35.47 ± 13.96	– CMAI-NH = 29.55 ± 8.89		
9	Traditional operas include sharing stories, demonstrating drama, singing, and memorizing songs, and performing	40 min/session, twice/week for 12 weeks	MMSE = 11.52 ± 3.39^ns^	11.74 ± 1.78^ns^	MMSE = 11.45 ± 2.22	11.45 ± 2.22
10	Listening, singing, discussion, games	60 min/session, twice/week for four weeks	CSDD = 5.6 ± 4.7*	CSDD = 5.2 ± 4.6**	CSDD = 6.8 ± 6.6	CSDD = 11.7 ± 7.2

CSDD: Cornell Scale for Depression in Dementia; MMSE: Mini-Mental State Examination; CMAI-C: Cohen-Mansfield Agitation Inventory scale, Chinese community-version; CMAI-NH: Cohen-Mansfield Agitation Inventory-Nursing Home version; ^a^ Chinese Version of the Cornell Scale for Depression in Dementia; Value = mean ± standard division, ns non-significant, *p < 0.05, **p < 0.01, ***p < 0.001

The standard methods to evaluate the effect of music therapy on dementia were the Mini-Mental State Examination (MMSE), Cornell Scale for Depression in Dementia (CSDD), Cohen-Mansfield Agitation Inventory (CMAI), and agitation. All studies combined various methods to assess the impact of music therapy on dementia. In Table 4, we included only common strategies for more studies, as explained in the Methods section. However, MMSE was the most utilized tool for such applications. All selected literature except study #10 employs MMSE. The MMSE scores for studies #6–#9 were only applied at baseline stages. The overall lowest (10.99 ± 4.16) and highest (22.71 ± 6.47) scores of MMSE were recorded in baselines of the study #8 experimental group and study #4 control group, respectively. The follow-up outcomes in the experimental groups of study #1 and #3 revealed improvements in their MMSE test scores, whereas studies #2, #4, and #5 decreased scores when compared with baseline counterparts. For instance, in study #1, the mean MMSE scores of the experimental group were 1.44–2.69 points higher than those of the control group at Times 2, 3, and 4, which were statistically significant differences (*p* = 0.026, *p* < 0.001, *p* = 0.044, respectively, Table 4). GEE analysis of scores for the six major cognitive domains in the MMSE indicated that the mean registration score of the experimental group was 0.50 points higher than that of the control group at Time 2, a statistically significant difference (*p* = 0.006) and that the mean recall scores for the experimental group were 0.63, 0.92, and 0.79 points higher than those of the control group at Times 2, 3, and 4 (*p* = 0.014, *p* < 0.001, and *p* = 0.004, respectively; Table 4). Likewise, in study 3, for the experimental group, the scores of MMSE had a statistically significant increase after 16 weeks (*p* < 0.01) after four months, which affirms that the level of cognition in elders with dementia significantly increased after receiving group recreational activities. This could be explained by the fact that the patients in familiar leisure activities might enhance cognitive reserve and delay the pathological onset of dementia.

The follow-up was compared with their corresponding baselines. Furthermore, studies #1, #4, and #10 employed the CSDD test to measure the effect of music therapy on dementia. The CSDD scores for all these studies exhibited a decline in the follow-up stage of the experimental group. CMAI was also used in the studies of #6 and #8. The detailed outcomes of music therapy on dementia can be referred to in Table 4.

## Discussion

This systemic review assessed the effect of music therapy on dementia for older adults residing in China. After the successive screening processes, ten studies were selected to be analyzed in this study. This review sought to provide up-to-date information on the topic as it covers recent research progress in the last decade (2013–2023). The current review identified the importance of incorporating a combination of various intervention approaches and measurement methods while studying the effects of music therapy on dementia. Most studies selected for this review implemented additional interventions along with music therapy (Table 4), including color sound bell (#1), folk recreation (#3), walking (#6), movement (#8), stories and drama (#9), discussion and games (#10), etc. Studies #2 and #5 only applied singing and/or listening. Moreover, these studies' cognitive function test results did not significantly improve. However, further research is needed to conclude this idea. Previous studies that were not included in this review also used additional activities such as cooking [23], nature videos [24], meditation [25], and painting [26] with music therapy to treat patients with dementia.

All studies selected in this systemic review (Table 4) applied multiple methods to measure the effect of music therapy on dementia. However, we have included in Table 4 those measurement methods common to at least two studies. MMSE has employed 90% of the studies. MMSE is considered the best and the most known tool to generate the overall measurement of cognitive impairment for clinical, community, and research settings [27]. This method is preferred due to its simple administration process and high acceptance among healthcare professionals working with individuals with dementia. The MMSE assesses an individual's orientation, concentration, attention, verbal memory, naming ability, and visuospatial skills, with a possible maximum score of 30 points. The scoring system of MMSE is as follows: higher scores suggest better cognitive functioning, a score of 23 or lower may indicate cognitive impairment and a score of 18 or lower suggests severe cognitive impairment [28]. Accordingly, the MMSE score for all the studies in the experimental groups indicated a severe cognitive impairment; out of the nine studies that included MMSE in their cognitive analysis, only five of the studies measured for both the baseline and follow-up stages. Out of these, studies #1 and #3 showed increments in their corresponding follow-up MMSE scores.

According to a recent study [27], using the MMSE alone as a single-administration test may not be sufficient to identify patients at risk of developing dementia. Therefore, it is crucial to conduct a series of tests to determine the status of dementia and develop proper management of patients. To this end, the studies selected for this systemic review have incorporated other dementia measurement techniques, such as CSDD, CMAI, and agitation (Table 4).

The effects of music therapy on dementia can be assessed by comparing (1) baseline and follow-up, (2) experimental and control groups within the same study, and (3) a particular study with other similar studies. It is worth noting that music therapy can also be used to treat and potentially slow the progression of dementia to its severe stages. For example, in study #10, the experimental CSSD, which was 5.6 ± 4.7 in the baseline, remained 5.2 ± 4.6 after four weeks of follow-up. On the other hand, the control group showed significant progress, with the score increasing from 6.8 ± 6.6 to 11.7 ± 7.2. Overall, this review showed that music therapy significantly affects dementia by improving depression and anxiety scores or prolonging disease progression easily, which are substantially related to older people's mental health.

The current review has limitations, including heterogeneously in the intervention approaches and cognitive measurement methods between the selected studies. These factors have a substantial effect in analyzing the effect of music therapy on dementia. Due to the limited number of studies included in this systematic review and the limitations above, it is challenging to arrive at a concrete conclusion. Nonetheless, we believe this study can offer valuable and up-to-date information on the impact of music therapy on dementia. Moreover, it can be a foundation for future studies and reviews in this field.

## Conclusions

Our study concludes that music therapy has improved cognitive and psychiatric trends in people with dementia. Furthermore, a long-term MT intervention positively affects depression and anxiety. Moreover, music therapy intervention seems to improve the quality of life of dementia patients. Music therapy could improve verbal fluency and reduce anxiety, depression, and apathy in selected patients living with dementia, although there do not appear to be proven benefits on memory, daily function, or overall quality of life. More clinical trials are needed to allow for more definitive conclusions on the therapeutic value of music therapy to patients with dementia.

## Future research recommendation

Regardless of limitations, music therapy is an effective intervention treatment. For this reason, more medical involvement and procedures should be promoted to confirm the positive effect of music interventions. Second, most review studies have fewer participants, which cannot provide robust, randomized findings. Since each clinical practice procedure and method is varied, it is necessary to implement a standardized medical trial to measure patients' cognitive function and behavioral features. More longitudinal studies are recommended to explore the development and benefits of music therapy. More theoretical models for outcomes and results should be defined to treat dementia patients.

## Data Availability

Not applicable.
